# Oxidative Stress and HPV Carcinogenesis

**DOI:** 10.3390/v5020708

**Published:** 2013-02-12

**Authors:** Federico De Marco

**Affiliations:** Laboratory of Virology, “Regina Elena” Italian National Cancer Institute, Via delle Messi d’Oro 156, 00158 Rome, Italy; E-Mail: demarco@ifo.it; Tel.: +39-0652662521; Fax: +39-0652662505

**Keywords:** HPV, oxidative stress, ROS, RNS, DNA damage, oxidative DNA adducts, viral carcinogenesis, cancer initiation, cancer promotion, cancer progression

## Abstract

Extensive experimental work has conclusively demonstrated that infection with certain types of human papillomaviruses, the so-called high-risk human papillomavirus (HR-HPV), represent a most powerful human carcinogen. However, neoplastic growth is a rare and inappropriate outcome in the natural history of HPV, and a number of other events have to concur in order to induce the viral infection into the (very rare) neoplastic transformation. From this perspective, a number of putative viral, host, and environmental co-factors have been proposed as potential candidates. Among them oxidative stress (OS) is an interesting candidate, yet comparatively underexplored. OS is a constant threat to aerobic organisms being generated during mitochondrial oxidative phosphorylation, as well as during inflammation, infections, ionizing irradiation, UV exposure, mechanical and chemical stresses. Epithelial tissues, the elective target for HPV infection, are heavily exposed to all named sources of OS. Two different types of cooperative mechanisms are presumed to occur between OS and HPV: I) The OS genotoxic activity and the HPV-induced genomic instability concur independently to the generation of the molecular damage necessary for the emergence of neoplastic clones. This first mode is merely a particular form of co-carcinogenesis; and II) OS specifically interacts with one or more molecular stages of neoplastic initiation and/or progression induced by the HPV infection. This manuscript was designed to summarize available data on this latter hypothesis. Experimental data and indirect evidences on promoting the activity of OS in viral infection and viral integration will be reviewed. The anti-apoptotic and pro-angiogenetic role of NO (nitric oxide) and iNOS (inducible nitric oxide synthase) will be discussed together with the OS/HPV cooperation in inducing cancer metabolism adaptation. Unexplored/underexplored aspects of the OS interplay with the HPV-driven carcinogenesis will be highlighted. The aim of this paper is to stimulate new areas of study and innovative approaches.

## List of acronyms used

**Table:** 

Acronym	Extended name
APE/Ref-1	(Human) apurinic (apyrimidinic) endonuclease/redox-factor
AP-1	Activator Protein 1
ATM	Ataxia telangiectasia mutated (protein)
ATR	ATM–Rad3-related (protein)
BaP	Benzo(a)pyrene
BER	Base excision repair complex
CAT	Catalase,
CDK1	Ciclin dependent kinase 1
ε-A	1N^6^ etheno-adenine
ε-C	3N^4^ etheno-cytosine
ETC	Electron transport chain
FAD	Flavin adenine dinucleotide
FapyAde	4,6-diamino-5-formamidopyrimidine
FapyGua	2,6-diamino-4-hydroxy-5-formamido- pyrimidine.
GSH	Glutathione
GPX1	Glutathione peroxidase 1
GSTP1	Glutathione S transferase P1-1
HIF-1	Hypoxia inducible factor - 1
HNE	*trans*-4-hydroxy-2-nonenal (HNE)
H_2_O_2 _	Hydrogen peroxide.
HPV/HR-HPV	Human papillomavirus /high-risk human papillomavirus
LPO	Lipid peroxydes
MAPK/ERK	Mitogen-activated protein kinase/Extracellular signal-activated Kinase
M2PK	M2 pyruvate kinase
NAD/NADH	Nicotinamide adenine di nucleotide/reduced
•NO	Nitric oxide
iNOS	Inducible nitric oxide synthase
8-oxoAde	8-oxo-7,8-dihydroadenine
8-oxo-Gua	8-hydroxyguanine, 8-hydroxy-2’-deoxyguanonsine
^1^O_2_	Singlet oxygen
O_2_•^− ^	Superoxide ion
RNS/ROS/RONS	Reactive nitrogen/oxygen/oxygen and nitrogen species
SESN	Sestrins
SESN2	Sestrin 2
SOD	Superoxide dismutase
Tg	5,6-dihydroxy-5,6-dihydrothymine (thymine glycol)
TGFβ-1	Tumor growth factor β-1
TopBP1	DNA topoisomerase II beta-binding protein 1
TRX	Thioredoxine reductase
VEGF	Vascular endothelial growth factor

## 1. Introduction

Cervical cancer is one of the leading causes of cancer death among women. The identification of HR-HPV infection as the etiologic agents of cervical cancer, as well as other non-genital carcinomas, is undoubtedly among the greatest medical advances that have been made in the last twenty years [1]. According to a widely shared view, these viruses must induce an enhanced proliferative state in order to accomplish their replicative cycle in the poorly replicating host epithelial cell. This is achieved through the activity of two oncogenic proteins, E6 and E7. Their ability to target and promote degradation of p53 and pRB, respectively, is believed to be the main mechanism by which HPV oncogenes induce genomic instability and allow the cells to acquire accumulating genomic alteration, thus ultimately leading to the full neoplastic state. However, only a minor percentage of viral infections lead to invasive growth, thereby indicating that viral oncogene expression is not *per se* sufficient, and other events are needed for cancer to occur. These additional events could be either the consequence of long-term viral protein expression, or the results of non-viral factors. In the search for such co-factor(s), many viral, host, and environmental factors have been investigated [2]. The role of oxidative stress (OS), however, has received little attention in this regard. OS is a condition occurring whenever the generation of oxidant species (mostly reactive oxygen species (ROS) and reactive nitrogen species (RNS), collectively known as RONS) exceeds the cellular neutralizing/scavenging capabilities. ROS are constantly generated in aerobic cells by the incomplete reduction of molecular O_2_ to H_2_O during mitochondrial oxidative phosphorylation, as well as during microsomal and peroxisomal oxidations. In addition, RONS are also generated during a number of processes such as inflammation, infections, and immune reactions [3], mechanical and chemical stresses (e.g. transient metal ions)[4,5], chemical biotransformation [6], exposure to UV [7,8], and ionizing irradiation [9].

Epithelial tissues, the elective target for HPV infection, are heavily exposed to all named sources of OS. RONS, through oxidative damage, cause functional alteration of cell membrane lipids, proteins, and nucleic acids [10]. In addition, through the perturbation of the cellular redox balance, they induce the activation of several redox-sensitive transcription factors, modify the gene expression responses, and modulate the function of redox-sensitive proteins. They have therefore been implicated in various acute or chronic degenerative processes, including aging and cancer [11,12,13]. Due to their conspicuous impact on cell homeostasis, RONS levels are closely monitored by sophisticated sensing mechanisms and are strictly controlled by multiple antioxidant and scavenging systems. Moreover, they are embedded with crucial roles within multiple cell-signaling and regulation pathways [14,15,16], having either pro- or anti-proliferative potential effects. Thus, OS represents an interesting candidate as a co-factor in HPV carcinogenesis. This paper was designed to provide a brief description of RONS generation and their biological impact, to summarize available data on the impact OS has on the major stages of HPV-mediated carcinogenesis, and to promote the identification of new areas of study and innovative approaches.

## 2. Generation of RONS

Mitochondrial respiration is a fascinating biochemical process operating a coordinated four-electron reduction of O_2_ to H_2_O. Although highly efficient, the mitochondrial electron transport chain (ETC) is imperfect [17] and leakage of intermediate products represents the major source of OS in aerobic organisms. The following brief description of RONS generation during mitochondrial respiration offers an outline of the basic phenomena and of their chemical properties. The contribution of other sources of RONS will shortly be mentioned in order to complete the background. 

*Generation of the superoxide ion.* The single electron (e-) reduction of molecular oxygen generates the radical superoxide ion (O_2_•- ) [18], according to the reaction (1).

O_2_ + e- → O_2_•- (1)

This radical species is also produced within the cell by the action of some oxidases such as aldehyde oxidase, NADH oxidase, and xanthine oxidase, as well as under UV irradiation. Although O_2_•- has the potential to react with almost all biological molecules, its direct contribution to cell damage is scarce, owing to its limited rate of diffusion and its comparatively low reactivity. Nonetheless, O_2_•- is able to sustain other cellular reactions generating extremely powerful RONS accountable for most of its toxicity. With the reaction (2)

O_2_•- + 2e- → ½ O_2_ + ½ H_2_O_2_ (2)

superoxide is rapidly dismutated (either spontaneously or enzymatically) to molecular oxygen and the much higher reactive hydrogen peroxide (H_2_O_2_). H_2_O_2_ and O_2_•- can further react according to the reaction (3), the “Haber–Weiss reaction,”

O_2_•- + H_2_O_2_ → O_2_ + OH- + OH• (3)

to generate the most reactive species hydroxyl radical (OH•). A further ROS, named singlet oxygen (^1^O_2_), is also generated as a by-product by the superoxide ion through the reactions (2) and (3) and by superoxide interaction with OH• [19]. Singlet oxygen is an excited form of molecular oxygen in which the electrons in the external shell are rearranged to confer increase oxidative activity. ^1^O_2_ attacks the double bond in lipids with formation of hydroperoxides and endoperoxides. It also attacks and oxidizes methionine, tryptophan, histidine, and cysteine.

*Generation of hydrogen peroxide*. In addition to being generated by the divalent reduction of O_2_ and protonation during electron transports (reaction 4)

O_2_ + 2e- + → H_2_O_2_ (4)

and through the above reaction (2), H_2_O_2_ is the primary product of many microsomal and peroxisomal oxidases. This non-radical ROS, with an intermediate reactivity between those of O_2_•- and OH•, is involved in thiol groups oxidation/inactivation and in redox potential imbalance [20]. Moreover, through the reaction (5), the “Fenton reaction”

H_2_O_2_ + Me^n+^ → Me^(n+1)+^ + OH- + OH• (5)

transition metal ions can catalyze the H_2_O_2_ reduction to OH- plus OH• and oxidized metal ion. The transition metal ions Me^(n+1)+^ can then be reduced back to Me^n+^ by the superoxide anion with the reaction (6)

O_2_•- + Me^(n+1)+^ → O_2_ + Me^n+^ (6)

This reaction, on one hand, scavenges O_2_•- equivalents, thus demonstrating a potentially beneficial effect, but, on the other hand, it reconstitutes the pool of reduced metal ions, thus allowing a further cycle to the Fenton reaction and further generating hydroxyl radicals.

The hydroxyl radical (OH•) is also formed under γ and x irradiation by direct lysis of water according to the reaction (7).

hν + H_2_O → OH• + H• (7)

**Figure 1:**
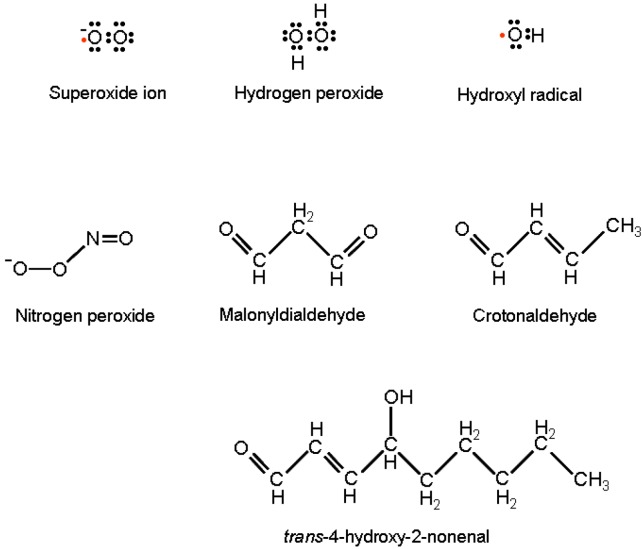
Lewis formulas of reactive oxygen and nitrogen species and structural formulas of lipid peroxidation products responsible for DNA modifications with miscoding potentials.

OH• is the strongest oxidizing species; it reacts with almost all biological molecules with rates close to the theoretical diffusion limit. It gives place to reactions of hydrogen extraction, hydroxyl addition, and electron transfer. OH• is the major cause of oxidative adducts on purine and pyrimidine bases, as well as on the DNA sugar moiety, resulting ultimately in nucleic acid point mutation, cross-linking and strand interruption. The hydroxyl radical further concurs to DNA modification by the activity of lipid peroxidation products (LPO) [21]. The most relevant LPOs are: malonyldialdehyde, Crotonaldehyde and trans-4-hydroxy-2-nonenal (HNE) (Figure 1).

These are generated by OH• hydrogen extraction from unsaturated lipids in membranes and, following their direct purine attack, are able to produce exocyclic DNA adducts with high miscoding potentials [22].

The nitric oxide (•NO), a radical RNS, is produced endogenously from L-arginine, oxygen and NADPH by several nitric oxide synthase (NOS) enzymes in response to a number of physiological stimuli. •NO is a very highly reactive species. With its short lifetime (a few seconds) and its ability to diffuse freely across membranes, •NO represents the ideal molecular messenger. As a paracrine or autocrine signaling molecule [23], it and is involved in the regulation of a number of functions including blood pressure, endothelial and neuronal homeostasis, platelet and leukocyte adhesion, phagocytes and antimicrobial activity, immune response, apoptosis induction. According to the reaction (8)

•NO + O_2_•- → OONO• (8)

nitric oxide, in the presence of superoxide generates the radical species peroxynitrite (OONO•) a highly reactive RNS itself able to induce lipid peroxidation and protein nitration [24].

### 2.1. Peroxisomal β-Oxidation

Another major source of RONS is the peroxisome fatty acid beta oxidation which generates H_2_O_2_ as a by-product. [25]. Despite the high concentration of catalase in peroxisomes, H_2_O_2_ leakage appears to be a physiological occurrence contributing significantly to OS as indicated by a direct correlation between the number of peroxisomes and their proliferation rate with the level of total cell OS and damage [26]. Moreover, the reported occurrence of catalase-negative peroxisomes during experimental liver regeneration [27] suggests that H_2_O_2_ leakage may be enhanced during rapid cell proliferation [28].

### 2.2. P450 Isoenzyme Activity

Microsomal cytochrome P-450 enzymes metabolize xenobiotic compounds, usually drugs or alkaloids of plant origin, by catalyzing their univalent oxidation or reduction. Depending on which P450 isoenzyme is involved, these reactions consist either in the direct reduction of O_2_ to O_2_•- [29] or in the single electron reduction of organic intermediates and the subsequent electron transfer to O_2_ with generation of O_2_•- and organic intermediate restoration [30].

### 2.3. Inflammation

Inflammation increases the risk of cancer development and, at the same time, is a constitutive trait of any cancer tissues, thus cancer itself represents a cancer-promoting agent [3]. During inflammation, the combined action of hormones, cytokines and low molecular weight messengers, the so-called “Go” signals lead to the recruitment of mast-cell and leucocytes [31]. These cells, upon a sharp increase of oxygen consumption, “the respiratory burst”, give way to a massive RONS release, including O_2_•-; H_2_O_2_; •NO and hypochloric acid (HClO) [32,33], at levels largely above the toxic threshold [3].

### 2.4. Ischemia–Reperfusion Stress

In addition to its central role in ischemic conditions such as myocardial infarction and brain stroke, ischemia-reperfusion stress is, less obviously, a relevant mechanism in peripheral areas of proliferating lesions. OS represents a major pathogenetic momentum in ischemia-reperfusion and, surprisingly enough, its generation requires both hypoxia/anoxia and reperfusion phases [34]. During ischemia–reperfusion, OS is generated by many different mechanisms, namely: leukocyte recruitment and respiratory burst; activation of xantine oxidase [35,36]; membrane NADPH oxidase activation [37]; mitochondrial impairment with increased RONS generation [38,39]; down-regulation of endogenous antioxidant mechanisms, as a consequence of mitochondrial hypoxia adaptation [34]; and, HIF-1-mediated induction of iNOS [40]. Altogether, these mechanisms have the potential to generate, directly or as secondary products, all types of RONS.

### 2.5. Signaling Pathways Activation

RONS are also generated during the activation of a large number of signaling pathways induced by growth factors, cytokines and tumor promoters such as: TGF alpha; TNF alpha; TGF Beta-1, *etc.* [39]. In turn, RONS can activate or potentiate many of these signaling pathways, often realizing a positive feedback circuit which further increases RONS generation [41].

## 3. Biochemical Effects of RONS

### 3.1. Genetic Damage

**Figure 2:**
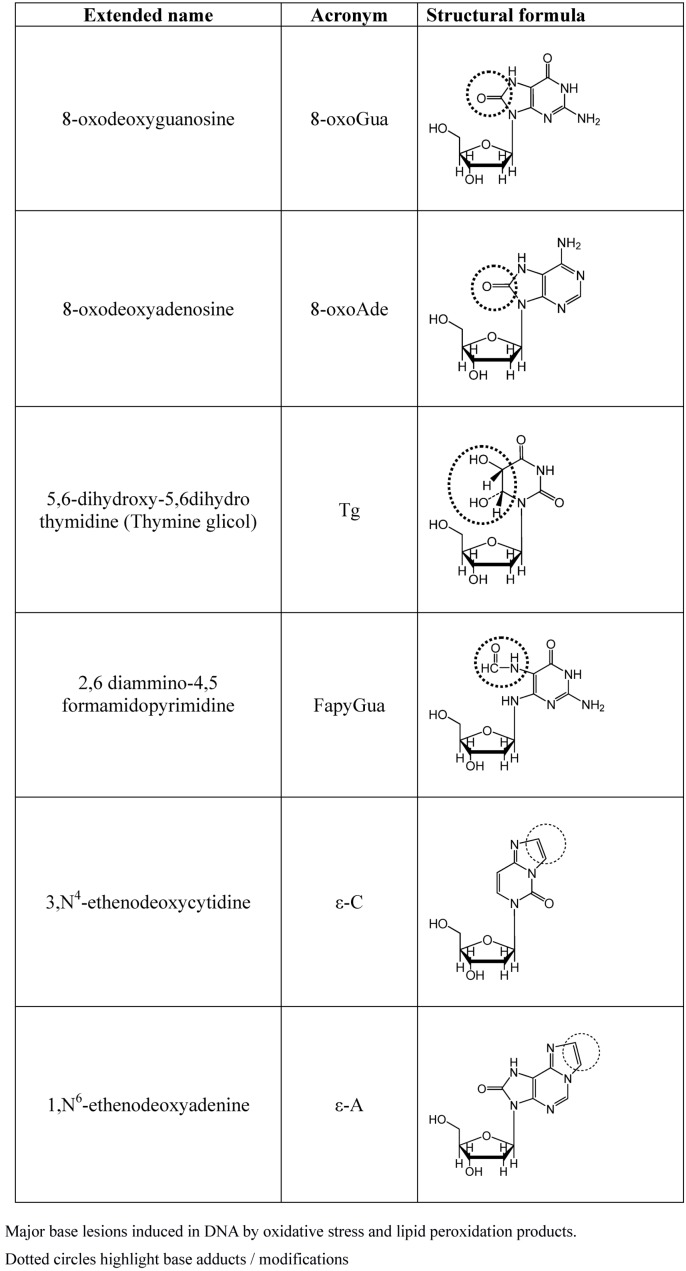
Major base lesions induced by OS.

More than one hundred oxidatively induced DNA lesions have been reported so far, and the current number may reasonably be even larger [42]. However, among them the most relevant products are those reported in Figure 2, namely 8-oxo-7,8-dihydroguanine (8-oxoGua), 8-oxo-7,8-dihydroadenine (8-oxoAde), 5,6-dihydroxy-5,6-dihydrothymine (thymine glycol, Tg) and the ring-opened lesion 2,6-diamino-4-hydroxy-5-formamido-pyrimidine (FapyGua).

All of these have high miscoding potentials [43,44]. Modified bases on DNA may result either by a direct oxidative attack on DNA or by incorporation of modified bases by polymerases. Indeed, free mononucleotide or bases are two to three orders of magnitude more susceptible to oxidative modification than incorporated bases. Although polymerases can discriminate regular and modified bases, this ability to discriminate is incomplete, and damaged nucleotides are incorporated with still relevant rates [45,46]. The major cause of DNA base modification is the direct attack by OH• and LPO products, mostly malonyldialdehyde, crotonaldehyde and *trans*-4-hydroxy-2-nonenal (HNE) is another one. These aldehydes, by direct DNA interaction, generate a number of DNA etheno-adducts such as 1N^6^ etheno-adenine (ε-A) and 3N^4^ etheno-cytosine (ε-C) [47,22]. Once inserted into the DNA helix,ε-As are responsible for inducing AT→GC, AT→CG and AT→TA point mutations [48], while the ε-Cs generates the mutation CG→AT, CG→TA and CG→GC [49]. In addition, LPO lead to the formation of DNA–DNA and DNA–proteins cross-linking, thereby causing chromosome breaks and rearrangements, in addition to gross structural aberrations [50,51].

### 3.2. Protein Damage

It is clear that DNA is not the only molecular target of OS. Proteins are obviously prone to oxidative modification as well, and a number of oxidative modifications have been described [52]. The most relevant protein oxidative modifications are carbonyls and nitro-tyrosine adducts. These adducts have been shown to increase in a variety of conditions, including ag,ing and cancer, and are currently used to measure the levels of protein oxidation [53,54]. Protein oxidation is mostly due to the direct attack of OH• and to a smaller extent due to the attack of malonyldialdehydes and HNE, while H_2_O_2_ accounts for a very minor part of it. Oxidized proteins are not repaired and must be removed through proteolytic mechanisms. The accumulation of oxidatively modified proteins in the cell lead to altered/disrupted functions, which can obviously induce, concur, or potentiate the homeostatic derangement which is the ultimate cause and the hallmark of cancer [55,56].

### 3.3. Modulation of Protein Function

In addition to the structural alterations on DNA and proteins, RONS can interact with the carcinogenic process by modulating the gene transcription and the signal transduction pathways. The NF-kB; Nrf2 are two major cancer-relevant systems modulated by RONS [57,58] and, reasonably, most of the cell pathways are regulated by, and depend on, oxidant signals [41]. Generally, the free radicals •NO and O_2_•^−^ act as direct signaling factors; H_2_O_2_ represents the major oxidant signal [59], and signaling occurs via oxidative activation/inactivation of phosphatases or transcription factors, activation of protein kinases; redox dependent modifications of thiol groups, *etc.* [59,60,61]. The modulation of multiple signaling pathways by RONS and the complementary RONS generation following activation of the signaling pathways has been extensively revised elsewhere, and their potential impact on cancer initiation, cancer promotion, and cancer progression adequately remarked upon [34,30,39,62].

In conclusion, aerobic life implies the generation of RONS. Due to their important physiological functions and sharp toxic activities, RONS represent major carcinogenic agents. It is widely accepted that HPV infection, through the expression of the E6 and E7 proteins, promote the degradation of the cellular proteins p53 and pRB, thus creating the conditions for the accumulation of cancer permitting genetic alteration. Thus, two different contributions of OS to HPV-driven carcinogenesis can be hypothesized: i) the mutagenic potentials of OS cooperate nonspecifically with the transforming activity of an HR-HPV infection. Such an additive mode can have important practical consequences, such as a shorter latency period or a higher efficiency in cancer induction; however, it does not imply any specific mechanism, rather, it represents a particular form of co-carcinogenesis; ii) OS can specifically modulate, or activate, or suppress, directly or indirectly, one or more HPV-specific molecular mechanisms orientating the viral/cell unit toward the transforming–carcinogenic process. The evidence about the latter hypothesis will now be reviewed and discussed.

## 4. The Interplay Between OS and HPV Carcinogenesis

### 4.1. OS and HPV Infection

It is commonly assumed—although not proven—that cellular conditions at the early steps of viral infection (i.e. viral adsorption, the viral entry and the initial establishment of viral expression) represent critical events for the outcome of HPV infection: spontaneously healing, productive infection *versus* persistent infection, and neoplastic transformation. The first data suggesting a possible involvement of OS in these early phases initially came from indirect clinical–epidemiological observations: in a study aiming to identify additional conditions for cervical cancer, the concentration of diamines and polyamines was evaluated in 187 seminal plasmas and the concentration of diamine oxidases and polyamine oxidases was evaluated in 126 cervical mucus samples. The seminal amines are oxidized by amine oxidases in the cervical mucus and the hydroxyl radical, hydrogen peroxide and LPOs are generated. The results indicated that highly variable concentrations of amines and enzymes were found in seminal plasmas and mucus, respectively [63]. It was therefore proposed that a highly divergent local burden of ROS and LPO can occur during sexual intercourse, and this variability could contribute to the fluctuating outcome of the viral infection. A recent perspective study correlating the clearance of incident HPV infection with the level of ferritin and of soluble transferrin receptor in 327 serum samples indicated that women with high levels of ferritin were less likely to clear incident HR-HPV infections than those with lower levels [64]. The authors suggested that elevated levels of iron storage may, by generating greater endogenous levels of ROS, be related to a reduced clearance rate. Along the same lines, Safaeian *et al.*, in a very large population-based study [65], reported that the polymorphism in gene *PRDX3* (a mitochondrial peroxiredoxin putatively involved in antioxidant activity and proliferation control) is associated with higher persistence and higher progression risk. Molecular data consistent with the above indirect evidences were recently reported. In a very interesting study on viral replication in organotypic “raft” viral cultures, it was shown that, following HPV16 infection, complete viral progeny assembly is dependent on a tissue spanning redox gradient. Exposition to both reducing and oxidant condition is sequentially needed for infectivity maturation and redox balance modulation in the cell microenvironment and sharply affects the infectious titer of viral progeny [66]. These results provide a molecular link connecting the OS with viral titer, a major factor potentially involved in persistent infection establishment, proposing a first mechanistic hypothesis for the connection between the oxidative status and lesion persistence/progression.

### 4.2. OS and Viral DNA Replication

After the viral infection, the core points of carcinogenesis are efficiency and accuracy of DNA replication. OS can strongly affect both, eventually creating the conditions for efficient viral integration. The first observation connecting OS with DNA papillomavirus replication came at the early beginning of the HPV and cancer story. In a BPV/mouse cell system, it was reported [67] that OS, either chemically or UV induced, was associated with amplification of both episomal and integrated copies of BPV. Such an amplification was linked to the DNA-damaging activity of OS, and to the occurrence of OS at the moment of viral infection, as no effect was produced by OS occurring after the infection, as well as by agents inducing cell proliferation (i.e. tumor-promoting agents) [67]. This initial observation was later extended with the report of a functional interaction between the TopBP1 protein of the base excision repair complex (BER) and the viral transcription/replication factor E2 enhancing the ability of the E2 protein to activate viral transcription and viral genome replication. [68]. Subsequently, the involvement of p53 in BER machinery sensing and repairing oxidative DNA damage was reported [69], and that in the presence of the E6 protein, the DNA repair functions are compromised and an increased 8-oxoGua accumulation takes place [70].

**Figure 3:**
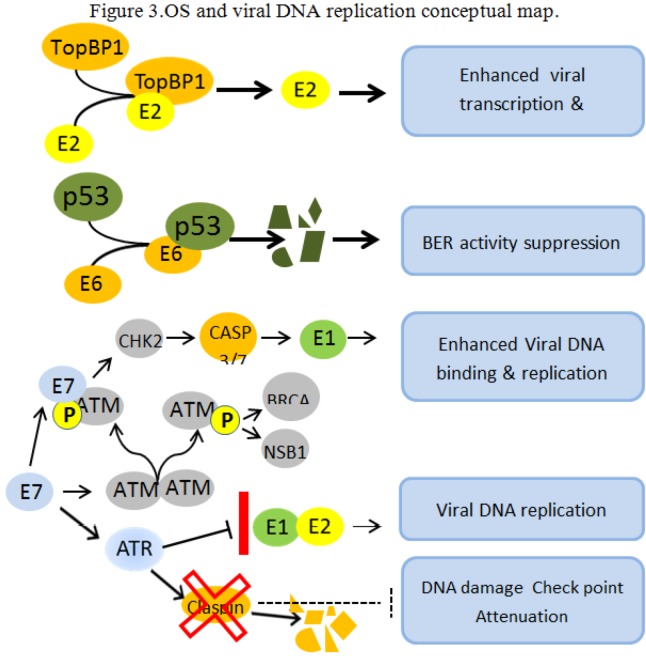
OS and viral DNA replication conceptual map. OS and viral oncogenes concur to create a cellular environment conducive to viral genome amplification and integration. E2 and TopBP1 interact *in vitro* and *in vivo* and TopBP1 enhance the ability of E2 to activate transcription and replication [68]. The E6-dependent p53 degradation abolishes BER functions leading to unrepaired cellular genome [69,70]. The E7 protein promote the phosphorylation of the inactive ATM dimer causing its dissociation into phosphorilated active monomers. The E7 protein then physically interact with the activated ATM monomer activating a number of proteins including CHK2, BRCA1 and NSB1. CHK2 induce proteolytic activation of Caspase 3/7, cleaving the E1 protein, and enhancing the E1 binding to its origin and its ability to replicate in an E2-independent manner [71]. The E7 oncoprotein attenuates the DNA damage checkpoint response by accelerating the proteolytic turnover of claspin, a critical regulator of the ATR/CHK1 signaling axis, in the G2 phase of the cell cycle [72]. These conditions permit the formation of abundant regular and rearranged viral copies, while multiple nicks and DS breaks in the host genome are unrepaired, thus creating the mechanistic feasibility for multiple viral integrations.

The connection between DNA damage sensing and HPV replication was further strengthened by the report of Moody and Laimins [71], showing that the activation of the ATM pathway, through the interaction with the E7 protein, specifically enhance the E1 DNA-binding and replication activity in an E2-independent manner. In addition, the E7 protein interacting with the ATR DNA damage pathway are able to alleviate the DNA damage checkpoint control by increasing the proteolytic degradation of claspin [72]. Finally, it was recently demonstrated that the E1 and E2 HPV-specific DNA replication is not arrested by ATR following DNA damage, both *in vitro* and *in vivo,* while SV40 replication is [73]. This lack of inhibition allows the HPV DNA replication to proceed in the presence of conditions inducing DNA double-strand breaks, thus strongly promoting viral integration (see Figure 3).

Taken together, the data above outline an OS-driven putative mechanism consistent with the viral DNA amplification and integration observed in cell lines derived from invasive cancers [74]. Namely, the OS activation of DNA damage pathways in the presence of HPV expression potentiates the activity of both E1 and E2 viral elements of DNA replication complex. In the presence of DNA damage, the viral replication proceeds, resulting in viral expansion and in increased production of rearranged and DS-break forms. The concomitant ATR-E7 dependent claspin degradation reinforce the release of the cell cycle checkpoint due to the p53 suppression and unrepaired cellular DNA is replicated. Thus abundant viral copies, including linearized and rearranged forms, are made available together with multiple nicks and DS breaks in the host genome, creating the mechanistic feasibility for multiple viral integrations.

### 4.3. OS and Apoptosis Suppression in HPV-Expressing Cells

Apoptosis suppression is a fundamental mechanism for cancer development, permitting the survival of transformed clones and their selection on the basis of their fitness to the specific microenvironment. HPV-transformed cells achieve this goal expressing the E6 viral protein that promote the ubiquitination and the proteolytic degradation of the p53 cellular protein. A major contribution to the modulation of apoptosis is provided by the activation of the inducible form of nitric oxide synthase (iNOS), directing the production of •NO. As mentioned above, •NO is a powerful chemical messenger and cell regulator with a double-edged pro-survival and pro-apoptotic effect. This seemingly contradictory profile, however, can be resolved by considering the level of •NO concentration involved in each specific condition and the temporal–spatial mode of its release. At high concentration, *i.e.* above 10^−6^ M, a concentration easily attained during the macrophage/neutrophils activation, •NO induces apoptosis by nitrosating thiol groups of cellular proteins and enzymes [75]. Conversely, •NO concentrations around 2 × 10^−8^ M are essentially mutagenic [76] and induce the release of vascular endothelial growth factor (VEGF) [77,78]. Thus, even though initially considered a potential mechanism of neoplastic growth control, it is now evident that •NO fails to attain apoptosis-inducting concentration in the vast majority of cancers of any histological origin [75]. In the case of cervical carcinomas and HPV-positive dysplastic lesions, the reason for the inadequate level of •NO is the insufficient expression of iNOS that appears to be progressively reduced with the histological severity of lesions [79,80]. This is the result of the TGFβ-1-mediated suppression of the iNOS expression at the mRNA level [81,82]. Accordingly, the level of TGFβ-1 mRNA observed in cervical smears correlates with the progression of cervical intraepithelial neoplasia to cancer [83]. Meanwhile, the low level of •NO concentration promotes mutagenesis, that further accelerates the neoplastic progression, and promotes the VEGF mediated angiogenesis that provides optimal metabolite supply to the tumor growth. Moreover, the increased blood flow potentiates the •NO scavenging by circulating red blood cells [84], thus completely arresting tumor apoptosis. Finally, E6 proteins, in a p53-independent way, specifically up-regulate the activity of the VEGF promoter, thereby inducing a high level of VEGF mRNA expression [85]. 

Although the suppression of p53 functions is a pivotal event in HPV carcinogenesis, it has to be remarked that the HPV E6 expression in the context of its natural promoter does not suppress p53 activity completely, which remains measurable both in cell lines [86] and in cancer-derived cells [87]. The extent of such a residual p53 function is inversely correlated to the E6/E7 and has the physiological role to maintain partial levels of the transcriptional activation, antioxidant systems, and DNA-repairing functions still necessary for the transformed cell survival [88]. The E6 expression level controlling the previously outlined mechanism of fine p53 down-modulation can be, in turn, modulated by a number of conditions [89,90,91,92], including OS that specifically modulates both the viral RNA expression levels [93] and their splicing pattern [94]. Thus, an OS-dependent modulation of the E6-p53 axis enables the cell to activate, when appropriate, a survival response consisting in a mild growth arrest, the appropriated activation of antioxidant enzymes and DNA repair mechanism, and a limited induction of apoptosis [93,94]. Altogether, the data provided earlier depict an E6/p53 interaction much finer than commonly perceived, consisting in a delicate, but incomplete, p53 suppression adjusted by OS to match the highly dynamic OS levels faced by the dysplastic/neoplastic cell.

Other specific cooperative connections between OS and HPV carcinogenesis are sustained by the E7 protein. This oncogene increases the resistance to H_2_O_2_-induced cell death through the up-regulation of catalase activity, the activation of NF-kB, and the orchestrated modulation of the cell factors: Bcl-xL; IL-18; Fas; Bad and Cytochrome-C [95]. In addition, E7, by specifically protecting the glutathione S transferase P1-1 protein (GSTP1) from oxidation, up-regulates the GSH-mediated OS detoxification and modifies the equilibrium between the oxidized and reduced GSTP1, consequently inhibiting the JNK phosphorylation and its ability to induce apoptosis [96]. Thus, the E6 and E7 proteins, in addition to their elective oncogenic roles, seem to provide the condition to survive the increased level of OS connected with the transformed status generated by them, turning a harmful, potentially limiting condition, into a positive selection factor. 

### 4.4. OS Cell Signaling and Metabolic Modulation of HPV Transformed Cells

A further OS-mediated regulation of viral oncogenes functions takes place at the transcription activation level. Early genes transcription is controlled by the E2 viral protein and by a wide array of cellular transcription factors [97]. Among them, a distinct role is played by the AP-1 complex. This is indeed a family of homo- or heterodimers composed of the variable association of two proteins among c-Jun; JunB; JunD; FosB; Fra-1 and Fra-2. In transformed or malignant cells with high levels of E6/E7 transcription, the AP-1 composition is mainly, but not exclusively, represented by the cJun/cFos heterodimer. However, the correct assembly of this powerful activating form is strictly dependent on the endogenous level of ROS [98] and, in conditions of low ROS levels, the variant complex cJun/Fra1 is preferentially formed. This form has a higher binding activity on the viral regulatory region and a repressive activity on transcription initiation and can therefore be associated with suppressed viral transcription and reduced oncogenic potentials. Accordingly, the cJun/Fra1 complex is preferentially expressed in non-neoplastic or reverting cell lines, while the canonical cJun/cFos form is expressed in oncogenic cell lines [99]. Conversely, the composition of the AP-1 complex seems to dictate the cell phenotype [100,101].

An elevated rate of aerobic glycolysis, the so-called “Warburg effect” [102], is a very striking metabolic feature of the cancer cell. A direct connection between the level of aerobic glycolysis in HPV neoplastic cells and the internal redox balance is provided by the physical interaction of HPV16 E7 protein with the glycolytic enzyme M2 Pyruvate Kinase (M2PK) [103]. M2PK is a regulatory enzyme occurring either in a high affinity tetrameric form or in a low affinity dimeric form. The balance of the two forms is controlled by the level of the key glycolytic intermediate fructose 1,6-biphosphate. The physical interaction with the E7 shifts the balance in favor of the dimeric form, inducing a sharp suppression of the tricarbossilic acid circle and oxidative phosphorylation, notwithstanding an elevated concentration of fructose 1,6-biphosphate. This suppression resulted in the depletion of the glycogen store and in the alteration of the redox balance between NADH and FAD [104]. The E7 protein is further able to induce an Na^+^/H^+^ exchanger-dependent alkalinization of the intracellular compartments that, presumably by modifying the activity of the many redox sensitive cytoplasmic proteins, directly modulate the cell proliferation rate, the growth rate in low serum, and the anchorage-independent growth induced by E7 [105].

### 4.5. Adaptation of Advanced Neoplastic Cells to OS

Data on OS in advanced cervical cancers has been steadily increasing since HPV infection was first proposed as a possible etiologic factor [106]. In a fairly large series of cervical histological samples, an increased level of oxidized protein thiol groups was found in cancer specimens compared to normal or dysplastic tissues, as well as compared to non-neoplastic areas surrounding the neoplastic lesion [107], therebysuggesting that highly oxidant conditions were acting on the cancer cells. Indications that HPV tumors experience an increased oxidative environment were then extended and confirmed by studies reporting on: an increased number of DNA adducts in histological samples forming invasive cervical cancers compared with dysplastic or normal tissue [108,109], the increased levels of thioredoxine reductase (TRX) and of apurynic apyrimidinic excision (APE)/Ref-1 in hypoxic micro-regions of cervical carcinomas [110], and by a growing number of reports correlating the activation or the increased expression of many other antioxidant enzymes with the dysplastic/neoplastic phenotype of cell lines or histological lesions [111,112,113]. However, some contradictory results were also reported [114]. Based on the reported results discussed earlier, the global oxidative status of patients has been also proposed as a potential index of efficacy in tumor treatment [115], or as a predictor of response to chemotherapy [116] or radiotherapy [117].

Indirect evidence also support the hypothesis that OS, in addition to being a hallmark of neoplastic growth, also has an active part in lesion progression. A large perspective cohort study showed that among women with a HPV-16 cervical infection, cigarette smoking, a powerful carcinogen with a wide range of different toxic activities, was associated with an increased risk of progression to CIN-III/invasive cancer [118]. Cigarette smoking, however, is also a known inducer of OS, which can be part of tobacco’s carcinogenic potential. This possible aspect is suggested by a parallel case-control study by the same group, reporting an inverse correlation of α–tocopherol plasma level with the risk of developing CIN and a protective role connected with the plasma levels of reduced ascorbic acid [119]. At the molecular level, benzo[a]pyrene (BaP), a major cigarette smoke carcinogen found in cervical mucus of women who smoke, has been shown to have a direct activity on HPV viral replication. Namely BaP, through a specific activation of the MAPK/ERK axis, activates CDK1, thus enhancing the titer of HPV16, HPV18 and HPV 31b once grown in organotypic “raft” culture [120]. These results propose a connection linking a non-mutagenic effect of BaP with the progression of HPV lesions, lending indirect support to the hypothesis that cigarette smoking may have a role as an OS inducer independent from its carcinogenic effect. Accordingly, data showed that antioxidant treatment (Vitamin C) of HeLa cells, through the modulation of the AP-1 composition, down-regulated the E6 expression and restored the p53-mediated apoptotic response to cisplatin and etoposide [121]. This suggests the possibility that antioxidant treatments could promote or restore tumor responsiveness to chemotherapeutic agents.

Complex data regarding advanced cancer and OS appear largely dispersed, somewhat recalling a patchwork of poorly related observations rather than an organic and consistent picture. Nonetheless, a direct correlation between an increasingly marked OS signature and the progression from mild dysplasia to severe dysplasia to invasive cancer is generally suggested by the majority of reports. Recent data, however, suggest a more articulated scenario indicating that, although an increasingly oxidant micro-environment is associated with tumor progression, tumor cells are characterized by a rather efficient control on RONS generation and oxidative damage attained by the appropriate up-regulation of antioxidant enzymes and detoxifying/pro-survival proteins [93,122]. This oxidative fitted modulation of cell metabolism turns the refractory, highly oxidant tumor environment into a positive selection factor for adapted cancer cells.

## 5. Conclusions

Theoretical consideration suggests OS and HR-HPV, two powerful initiating and promoting carcinogens, can act synergistically. Indirect clinical epidemiological evidence and initial biochemical data support the conclusion that viral infection, establishment of persistent-chronicle infection, and viral integration are indeed potentiated by OS. Both pro-survival and pro-apoptotic mechanisms have been described for OS; however, in viral dysplastic lesions, OS actually behaves as a pro-survival factor promoting the AP-1 mediated expression of E6 and E7, specifically reinforcing their anti-apoptotic mechanisms. A number of antioxidant enzymes and detoxifying pathways are consistently associated with HPV transformed cells that seem to be well equipped to fit highly oxidant environments. A number of questions, however, remain unanswered: No experimental evidence is available about the effect OS has on the regulation and functions of the E4, E5, L1 and L2 genes, as well as their proteins. Conversely, no data are available on the effect that E2, E4, E5 L1 and L2 viral proteins have on the OS generation, scavenging and repair. Apart from data on VEGF-mediated neo-angiogenesis, no conclusive data are available on the mechanisms involved in tissue invasion and remodeling, immune escape, cell shedding and metastasis homing. Only preliminary, non-systematic results are available on the role of OS in chemotherapy response/resistance and only initial attempts have been made to exploit OS signature as a potential marker for diagnosis and for clinical outcome of lesions. A large amount of work remains to be done.
